# New Indications for Liver Transplantation

**DOI:** 10.3390/jcm10173867

**Published:** 2021-08-28

**Authors:** Alberto Zanetto, Sarah Shalaby, Martina Gambato, Giacomo Germani, Marco Senzolo, Debora Bizzaro, Francesco Paolo Russo, Patrizia Burra

**Affiliations:** Multivisceral Transplant Unit, Department of Surgery Oncology and Gastroenterology, University of Padova, Via Giustiniani 2, 35128 Padova, Italy; alberto.zanetto@yahoo.it (A.Z.); sarahshalaby18@gmail.com (S.S.); martina.gambato@gmail.com (M.G.); germani.giacomo@gmail.com (G.G.); marcosenzolo@hotmail.com (M.S.); debora.bizzaro@gmail.com (D.B.); francescopaolo.russo@unipd.it (F.P.R.)

**Keywords:** alcoholic hepatitis, acute-on-chronic liver failure, cholangiocarcinoma, colorectal cancer metastases

## Abstract

Liver transplantation (LT) is an important therapeutic option for the treatment of several liver diseases. Modern LT is characterized by remarkable improvements in post-transplant patient survival, graft survival, and quality of life. Thanks to these great improvements, indications for LT are expanding. Nowadays, clinical conditions historically considered exclusion criteria for LT, have been considered new indications for LT, showing survival advantages for patients. In this review, we provide an updated overview of the principal newer indications for LT, with particular attention to alcoholic hepatitis, acute-on-chronic liver failure (ACLF), cholangiocarcinoma and colorectal cancer metastases.

## 1. Introduction

Since the first procedure performed in 1963, liver transplantation (LT) has become an important therapeutic option for the treatment of inborn metabolic disorders, acute liver failure, end-stage chronic liver disease, and primary hepatic cancers [1].

Over the past several decades LT has continued to grow and evolve with huge improvements in surgical techniques, organ preservation and procurement, and immunosuppression. Therefore, the modern LT is characterized by remarkable improvements in post-transplant patient survival, graft survival, and quality of life. Thanks to these ever-increasing improvements in overall survival, with one-year graft and patient survival nowadays around 90% [2], indications for LT are expanding, also as a result of a better understanding of liver diseases and innovative therapies.

**Figure 1:**
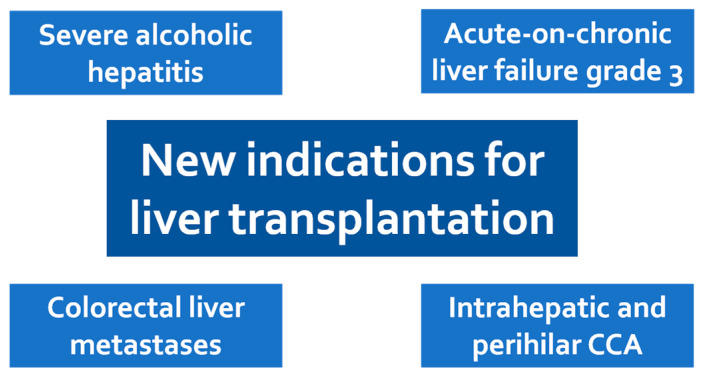
New indications for liver transplantation. *Controversial* indications include severe alcoholic hepatitis and ACLF grade 3. *Questionable* indications include non-hepatocellular carcinoma liver cancer, and liver metastases from colorectal cancer. CCA: cholangiocarcinoma; ACLF: acute-on-chronic liver failure.

Nowadays, clinical conditions historically considered exclusion criteria for LT, such as severe alcoholic hepatitis (AH), acute-on-chronic liver failure (ACLF), colorectal cancer metastases and cholangiocarcinoma are now considered new indications for LT, showing survival advantages for patients. In this review, we provide an updated overview of these newer indications for LT (Figure 1).

## 2. Alcoholic Hepatitis

Worldwide, alcohol-related liver disease (ALD) is one of the most prevalent liver diseases and the second most frequent indication for LT [3], representing around 30% of all primary LT procedures in Europe and approximately 25% in the USA [4,5].

The first cornerstone in the treatment of patients with ALD is abstinence from alcohol. An adequate time of abstinence may decrease hepatic fibrosis, reduce the risk of progression to cirrhosis, improve the prognosis of cirrhotic patients and reduce the mortality [6,7,8]. Abstinence is important, but usually it cannot reverse advanced ALD and in many cases the only definitive treatment for ALD is LT. Despite the fact post-LT outcomes and survival rates are analogous with those of other etiologies [4], ALD is still judged a controversial indication for LT. The discussion is generated mainly by the opinion that ALD is a self-inflicted disease, and by the possible risk of harmful effects to the graft after alcohol relapse [9]. In the majority of transplant programs, a period of 6-month of abstinence (“six-month rule”) is a compulsory condition to consider a patient eligible for LT. This rule has a double scope: first, to avoid LT in those patients in whom liver function and general clinical status will improve after alcohol removal, second to identify patients at higher risk of relapse after LT.

Nevertheless, the role of the pre-LT extent of abstinence as a predictor of alcohol relapse post-LT has not been clearly confirmed and the enforceability of this rule is still controversial [10]. Indeed, in a systematic review including 22 studies, in only two of them the six months of alcohol abstinence was predictive of post-LT relapse [11]. Furthermore, the ideal period of abstinence pre-LT is still controversial, although there are data confirming that a shorter prelisting abstinence period is associated with a faster post-LT relapse [12].

In recent years, an alarming increase in incidence of hospitalization for AH and mortality rates has been observed both in the US [13] and in Europe [14]. AH presents with fatigue, anorexia, nausea, jaundice, mild-to-moderate increase of transaminases, hyperbilirubinemia, hypoalbuminemia, elevation of neutrophils and prothrombin time (PT) prolongation [3]. The most used validated prognostic scoring system is the Maddrey Discriminant Function (MDF). Usually AH is defined by a MDF >32 [15]. The role of pharmacological treatments, especially corticosteroids, in patients with AH is still debated, with studies demonstrating efficacy in improving survival [16,17,18] and others showing a negligible effect on reducing mortality [19,20]. In patients not responding to medical therapy the prognosis is very poor, with a 6-month mortality rate of 75%.

In accordance with the “six-month rule”, AH patients are ineligible for LT at most transplant centers. Nonetheless, there is growing evidence that, in selected patients after the first episode of AH not responding to medical therapy, LT represents an effective treatment [21,22]. it was demonstrated that the post-LT outcomes are good, with survival rates significantly higher compared to not transplanted patients with AH not responding to steroid therapy [23,24,25].

Like with ALD cirrhotic LT recipients some ethical and social concerns remain. These are mostly originated by the public opinion that a graft is afforded to patients who were actively consuming alcohol immediately previous to admission on the waiting list, with higher risk of post-LT alcohol relapse. However, the existing data on LT in these patients demonstrate that relapse rates are analogous to those observed in patients with ALD that respected the “6-month rule”, if a rigorous and appropriate selection process is applied [23,24,25].

Multiple arguments for either “tight” or “loose” selection criteria have been proposed for LT in AH [26]. One major argument for tight selection is that current models for predicting survival without LT are not adequately precise for use in an individual patient, which implies not only that some patients will be subjected to LT unnecessarily, but also that others will be denied a potentially lifesaving LT. Additionally, most criteria for listing rely on clinical judgment, which may vary across different centers, thus leading to inequity of access to LT. On the other hand, real-life patients with AH undergoing LT often present with ACLF and a high risk of short-term mortality, thus making unnecessary LT very rare. Furthermore, a careful selection of patients at the first event of liver decompensation has repeatedly yielded excellent outcomes and low risk of relapse [26].

Like other indications for LT, further refinement of selection criteria is expected to evolve gradually over time. However, without the establishment of national and international agreement on criteria for admitting in waiting list and transplanting patients with AH, a high variability persists in terms of admittance to LT for those patients, with disparities that are manifest also at a national level with a potential inequality among patients with the same clinical conditions [27].

Data reporting good outcomes of early LT in selected patients were published in the last years. In the study by Mathurin et al. [23], 26 patients with AH with no response to corticosteroids were subjected to early LT as rescue therapy, after a strict multidisciplinary selection process. Survival after 6 and 24 months post-LT were significantly higher than in matched not transplanted controls (77% vs. 23%). Alcohol relapse was detected up to three years after LT in about 10% of patients.

A US study, published by Im et al. [28] confirmed the good outcomes of early LT in 94 patients with AH, in whom the 6-month survival rate was higher compared with matched not transplanted patients (89% vs. 11%). Alcohol relapse was diagnosed in only one recipient at 180 days after LT. Similarly, in a retrospective study published by Lee et al. [29], cumulative patient survival percentages after LT for AH were 94% and 84% at 1 year and 3 years, respectively After LT, 72% were abstinent, 18% had occasionally relapses, and 11% had sustained alcohol intake.

In Italy, Germani et al. coordinated the first Italian experience in a pilot study on early LT for AH from four different LT centers. Among those centers, the coordinating center is the Multivisceral Transplant Unit of Padua University Hospital. The inclusion criteria were AH, as a first episode of decompensation in chronic liver disease and no responses to medical therapies, but more importantly, the patient should have been socially integrated and have supportive family members, with psychiatric assessment and addiction profile and no comorbidities [30]. Preliminary data coming from Padua Liver Transplant center demonstrated a significantly higher survival rate amongst patients who underwent early LT compared to non-responding patients who were denied early LT.

The Spanish Society of Liver Transplantation has recently published a consensus statement on the potential expansion of indications for LT including patients with a first episode of severe AH not responding to medical therapy [31], whereas no specific guidelines or position statements have been published with this regards in Germany. In UK a pilot program for LT in patients with severe AH was developed. Over a 3-year period 20 patients aged between 18 and 40 years were evaluated, but none underwent LT, mainly due to the extremely stringent criteria for listing and the need for unanimity among members of the transplant panel [27].

The most significant concern in patients actively drinking before admission, is the post-LT risk of relapse. In the already mentioned landmark paper [23], about 10% of patients had a relapse up to three years after LT. This could be important not only from the “single-patient” perspective, but also for the possible negative effects on donation rate. Nevertheless, a recent multicenter survey suggests that organ donation was not negatively influenced by the early LT for AH [32]. Given the complexity of the selection and management of patients with AH, a multidisciplinary approach, involving various stakeholders including transplant hepatologists and transplant surgeons, but also psychiatrists, psychologists, and addiction specialists is becoming compulsory to accurately evaluate LT candidates [33,34]. The SALT prognostic score, developed including four objective pre-LT variables, was proposed in order to foresee the risk of sustained alcohol intake after early LT for AH assisting in the selection of patient candidates for early LT or in advising controls post-LT [35].

The psychosocial assessment of LT candidates and the evaluation of social background, including the presence of an active and effective support by the family, are essential parts of the pre-transplant evaluation process. In fact, the transplant outcome is undoubtedly influenced also by psychosocial and behavioral issues along with the usual medical factors [36]. This concept is even more important in the context of early LT where the psychosocial assessment is essential for the establishment of the real probability of long-term abstinence. Indeed, alcohol abuse is frequently associated with depression, personality disorders and other psychiatric disease, that can affect the post-transplant outcome of these patients [37,38]. 

To ensure to the LT candidates for AH the best long-term outcomes, globally accepted clinical and psychosocial selection criteria should be identified [39]. Very strict criteria should be explored for the early LT in this setting, as indicated in an Italian position statement [34]. Notably, a transparent and direct interaction between clinicians and society, based on the concept of no “a priori” exclusion to the evaluation for LT in the case of AH is essential.

## 3. Acute-On-Chronic Liver Failure (ACLF)

ACLF is a clinical syndrome characterized by acute decompensation (AD) of chronic liver disease, development of organ failures and systemic inflammation, and high risk of short-term mortality (>15% at 28 day) [40,41,42,43]. Development of ACLF in patients with chronic liver disease results from various precipitating factors that vary according to geographical regions: alcoholic hepatitis and bacterial infections in the West and relapse of chronic HBV infection in the East [44]. In approximately 40–50% of the cases, however, development of ACLF is not associated with identifiable triggers and current hypothesis in these cases is that metabolites from gut bacteria or translocation of DAMPs from leaky gut (or a combination of both) may be involved and trigger inflammation which in turn leads to organ dysfunction/failure [44]. There is no specific treatment for patients with ACLF, and current management include treatment of associated complications/precipitating factors and organ support. In patients with ACLF due to one or more specific factors (i.e., bacterial infections, alcoholic hepatitis, bleeding events, drug-induced liver injury), early identification of trigger factor(s) and specific treatments are important though it’s unclear whether this can really prevent worsening of ACLF [40]. All patients with ACLF should be preferably managed in a tertiary care center and by a multidisciplinary team including transplant hepatologists, ICU doctors, and transplant surgeons. General management of patients with ACLF and their complications should follow current guidelines for management of critically ill patients with cirrhosis [45]. Patients should be monitored frequently and evaluated serially for potential transfer to intensive care unit. Each organ dysfunction shall be treated specifically in order to prevent a stage in which multiorgan failure occurs and all treatments eventually become futile [45].

Per current consensus, severity of ACLF is defined by number of organ failures, and is not surprising that patients with three or more organ failures have increased risk of mortality compared with those with one or two organ failures. Specifically, patients with ≥3 organ failures have grade 3 ACLF (ACLF 3), and 28-day mortality in these patients approaches 80% [40]. Given such a high risk of death and the lack of alternative medical treatments, LT may be the only viable option in certain patients with ACLF [46,47]. Yet, selection of patients suitable for LT, prioritization of candidates during wait-list time, and best timing for LT in ACLF remain problematic [48]. Here, we discuss the current knowledge and the main open issues regarding LT in patients with ACLF, particularly those with ACLF 3.

### 3.1. Wait-List Priority in Patients with ACLF Awaiting LT: Beyond MELD-Based Allocation

ACLF is a rapidly progressive syndrome with a variable course [49,50]. On the one hand, it is important to identify patients with potential for full recovery, in whom LT would be unnecessary and not beneficial; on the other, one has to identify those at higher risk for progression, in whom development of either sepsis or irreversible organ failures can compromise eligibility for LT and post-transplant outcomes [41]. In these patients, the therapeutic window for LT is significantly narrow and unexpected clinical deterioration may determine removal of candidates from the waiting list [51].

Unfortunately, the discrimination between these groups remains unclear. The Model for End-Stage Liver Disease (MELD) score, that is used to estimate wait-list mortality and guide organ allocation in patients with cirrhosis awaiting LT, is not appropriate to predict survival in candidates with ACLF [52]. In fact, MELD does not reflect severity of hepatic encephalopathy and respiratory/circulatory failures, which are major drivers of mortality in ACLF [40,41,42]. Also, it does not include biomarkers of systemic inflammation, such as white blood cells and levels of C reactive protein, which reflect severity of ACLF and correlate with survival [53]. 

In a retrospective analysis based on the United Network for Organ Sharing (UNOS) database and including approximately 100,000 patients, Sundaram and coworkers demonstrated that the risk of death in ACLF 3 was 44% even if their MELD score was <25, and was greater than that in advanced patients as defined by a MELD >35 but without ACLF [53]. In an independent cohort including 71,894 veterans with decompensated cirrhosis, Hernaez et al. found that in those with ACLF the probability of 90-day mortality was significantly higher than the one predicted by MELD-Na alone [54]. In a third, large retrospective analysis including patients from the UNOS registry between 2002 and 2014, those with ACLF 3 (*n* = 5099) had a significantly higher risk of death at 14 days than those listed for acute liver failure (*n* = 3377), regardless of MELD-Na [55]. Taken together, these studies indicate that in ACLF patients, particularly grade 3 ACLF, an early discussion about LT should be initiated independent of their MELD status. 

Whether a combination of MELD score and grade of ACLF could be the optimal strategy to assess wait-list priority in patients with ACLF has not yet been thoroughly investigated. To this end, in a large, retrospective study including 18,416 candidates with ACLF from UNOS registry, Abdallah showed that the severity of ACLF and MELD score interacted synergistically in anticipating the risk of mortality at 90-day, and that the effect of ACLF grade was relatively more relevant at lower (i.e., ≤25) levels of MELD [56]. A new prognostic tool integrating MELD score and grade of ACLF was therefore proposed to mitigate disparities regarding organ allocation in ACLF, particularly for candidates with a MELD score ≤ 25 [56]. If these results will be confirmed in prospective cohorts, it is plausible that a combination of MELD score and ACLF grade will become the next standard to assess priority of candidates with ACLF. 

In conclusion, there is a strong need to improve the MELD-based allocation to mitigate wait-list mortality in candidates with ACLF [47,57]. Innovative scores are supposed to capture recipient factors (number and severity of organ failures), global patient’s status and performance (sarcopenia and frailty), and chronic associated conditions (comorbidities). This could lead to a more personalized approach regarding management of wait-list priority in ACLF and would ultimately improve patient’s survival and LT outcomes [58]. 

### 3.2. Benefit of Liver Transplantation in Patients with ALCF Grade 3

Patients with ACLF 3 have a 28-day LT-free survival of 20% [40,41,42]. Considering such a high risk of death, LT is a potentially life-saving treatment for the vast majority of these patients. Preliminary data, however, suggested that post-transplant survival in ACLF 3 might be lower than that in recipients transplanted for decompensated cirrhosis [59]. Hence, the question rose as to which all patients with ACLF, independent of ACLF grade, could be considered for transplantation [57]. 

Results from other studies, on the other hand, indicated good survival 1-year after LT [53,60,61,62]. Sundaram et al. retrospectively analyzed the UNOS database for the years 2004–2017 [63]. In total, 56,801 patients received LT and 54.6% had no ACLF, 15.4% had ACLF 1, 15.9% had ACLF 2, and 14.1% had ACLF 3. Interestingly, survival at 1-year post-LT was comparable between patients with ACLF 3 and those with grade 0–2, and that was above 80% in all groups [63]. More interestingly, although patients with ACLF 3 had lower long-term survival compared with those with grade 0–2, 68% of patients who received LT for ACLF 3 were alive 5 years after transplantation, which would justify both the transplant benefit in these patients, set at >50% 5-year after LT [64], and the acceptable utility of donor grafts. Comparable findings were reported by multicenter cohort studies from Europe [49,60,65] and by one recent study from Asia that first evaluated the outcomes of living donor LT for the treatment of ACLF [66]. In this study, including 321 candidates with high MELD score who underwent living donor LT, survival at 5-year was comparable between patients with and without ACLF (72% versus 81.82%), and 1 year-survival in ACLF 3 was comparable with that of grade 1 and 2 (76% vs. 85% vs. 93%; *p* = 0.2) [66]. Taken together, these studies indicate that ACLF grade 3 is not an absolute contraindication for LT, and that satisfactory outcomes can be achieved provided there is a good selection of candidates.

To this end, new methods to improve assessment of LT eligibility in these patients are eagerly awaited, that is to evaluate whether an individual patient has become “too sick to be transplanted” [58,67]. The fact that no patient with ACLF and severe respiratory failure in the CANONIC trial underwent LT indicated that this condition was considered an absolute contraindication for LT in ACLF [49]. Two large multicenter studies from US [53,68] indicated the following factors to be associated with higher risk of death post transplantation: need for mechanical ventilation at transplant, levels of lactate >4 mmol/L before transplantation, pre-transplant white blood cells count within normal limits, older age of recipient, and transplantation of marginal-grafts. The combination of three different organ supports (dialysis, vasoactive drugs, and mechanical ventilation) has been proposed as a potential criterion to withhold LT [51], however it may also prevent transplantation in a significant number of subjects with potentially favourable outcome. In fact, other factors may affect severity of organ failures and therefore the chance to perform a successful LT. This includes indications for organ support (i.e., ventilation for severe respiratory dysfunction vs. grade IV encephalopathy) and intensity (dose of vasoactives) and/or duration (i.e., 2–3 vs. >7 days) of organs support.

Given the increasing number of LTs performed in patients with ACLF worldwide, a better understanding of how to define too-sick-to transplant patients and thereby avoid “futile” transplantations is urgently needed [58]. Not only it is important to confirm whether LT in ACLF 3 confers a significant survival benefit, but also whether is associated with an improved quality of life [69]. For example, pre-transplant acute kidney injury (AKI), that is commonly observed in cirrhosis and ACLF [70,71,72], is a major predictor of post-LT chronic kidney disease (CKD) and need for replacement therapy [73]. Although the burden of CKD after LT in ACLF 1 may not be substantially increased [74], more specific results regarding patients with ACLF 3 are lacking. Long-term data regarding quality of life after LT for ACLF are awaited and may help to improve selection criteria and management of candidates with ACLF both before and after LT [75,76].

### 3.3. Timing of LT: The Earlier the Better?

One major challenge in candidates with ACLF is to assess the appropriate timing for LT. Given their high risk of death, one would expect that “as soon as possible” could lead to the greatest transplant benefit. In support of this assumption, in a landmark analysis of UNOS database, Sundaram and Jalan demonstrated that patients with ACLF who received LT within 30 days within listing had higher survival at 1-year than those who were transplanted thereafter (83% vs. 79%, respectively; *p* = 0.03) [53]. The same study demonstrated that LT within 30 days could significantly improve survival in patients who underwent LT on machinal ventilation (77% vs. 72%; *p* = 0.03). Comparable results were described in the CANONIC trial where survival at 6 months in patients with ACLF 2 and 3 who underwent LT within 28 days was 81% compared with 10% in those treated with medical therapy [49]. 

Yet, other evidence suggested that the benefits of early LT have to be balanced against benefits conferred by resolution of organ failures. In a retrospective trial including 98 candidates with ACLF who received LT, the 37 who had improvement of ACLF grade prior to transplantation had a significantly better survival compared with controls with no improvement [61]. Similar results were reported by Sundaram in a larger study from UNOS data [77]. The authors included 3636 candidates with ACLF 3 who received LT within 28 days of listing. Of these patients, 24.5% recovered to either no ACLF or grade 1 or 2 ACLF, whereas 75.5% remained with ACLF 3 at time of surgery. Interestingly, survival at 1 year was 82% in patients who underwent LT with ACLF 3 and 88% in patients recovering to ACLF 0–2 (*p* < 0.001). Furthermore, the probability of survival of ACLF 0–2 who worsened to ACLF 3 was significantly lower than in patients who remained at ACLF 0–2 (84% vs. 90%; *p* < 0.001). However, <25% of candidates with ACLF 3 at enlisting were able to achieve a lower grade of ACLF [77]. Hence, while in principle it would be optimal to undergo LT upon recovering of organ failures, this appears not doable in the major part of patients with ACLF 3. 

In summary, in candidates awaiting LT for ACLF 3, two major variables need to be balanced to assess the best timing for transplantation, that is on one hand the individual patient’s risk of progression and death on the waiting list, on the other whether there is any chance to postpone transplantation with the goal of waiting for ALCF to improve prior to LT [43,58]. 

## 4. Colorectal Liver Metastases and Liver Transplantation

Colorectal carcinoma (CRC) shows an incidence of about 700 per million population in Western countries and the liver is involved in approximately 70% of patients with colorectal metastases [78]. Currently, the only potentially curative treatment is represented by surgical resection of metastases [79], with a median 5-year survival of 30–40% compared to only 5% in those non-resected [79,80]. Despite the recent advancements in surgical techniques, only ~20% of patients are resectable at diagnosis [81] and the disease often recurs within 3 years of resection [82]. Percutaneous radiological treatments such as ablative treatments (radiofrequency, microwave and cryosurgical ablation, transcatheter intra-arterial therapy), hepatic arterial infusion chemotherapy, transarterial embolization and chemoembolization, and radioembolization with yttrium 90 can be applied to achieve tumor resectability. Even though it has been demonstrated that interventional radiology contributes to the improvement of overall survival rates [83,84], its role in the curative intent is still marginal. Palliative chemotherapy remains the main option for patients non candidable for surgery. Even though the initiation of first-line chemotherapy in selected patients with good performance status, no KRAS or BRAF mutations, and left-sided tumors prolongs median overall survival [85,86,87,88,89], prognosis remains poor and only ~10% of them survive up to 5 years [86,90]. Interestingly, recent clinical trials show an improvement of median survival with modern chemotherapy including the use of bevacizumab/EGFR antibodies, from 6 months to 2 years [85,91,92].

In this context, LT has been explored as an option to remove all viable disease in those patients with disease spread limited to the liver, which are not elegible for resection due to the low remnant liver volume [93,94]. In the past, several attempts were performed obtaining 5-year overall survivals <20% [95]. Due to these poor results and organ shortages, active colorectal liver metastasis remained a contraindication for LT thereafter. Nonetheless, the majority of these patients were disease-free at death, which was due to transplant-related complications instead. A prospective study from Norway a few years ago (the SECA-I study) has renewed focus for this potentially curative option. Patients included in this study had completed surgical resection of the primary tumor, had a good performance status, and received LT after at least 6 weeks of chemotherapy. The estimated 1, 3 and 5-year overall survival after LT were 95%, 68% and 60%, respectively. The median follow-up was 27 months (range 8–60 months) and disease-free survival was 35% at 1 year. The candidates with the best prognosis were those with presence of colorectal liver metastasis at diagnosis, pN0, pretransplant maximal CCR diameter <5.5 cm, levels of carcinoembryonic antigen (CEA) <80 mg/L, response or stable disease on chemotherapy, and >2 years from diagnosis to LT [93]. The 5-year overall survival rate of these patients with favorable prognostic factors was similar to that of patients transplanted for hepatocellular carcinoma (HCC) following the Milan criteria [96,97]. Interestingly overall survival was much longer when compared to disease-free survival. As a matter of facts, most recurrences in CRC patients were slowly-growing lung metastases amenable to treatment, regardless of immunosuppression [98,99], which is not the case for HCC-recurrence after LT. As a reinforcement to these results, LT was demonstrated to produce longer 5-year overall survival rates when compared to chemotherapy in patients non amenable to surgical resection of liver metastastases when data from the SECA-I study were compared to those of the NORDIC VII trial (56% vs. 9%, respectively) [88].

Following the SECA-I study, Toso et al. reported the results of 12 patients with colorectal liver metastases undergoing LT, confirming 1-, 3- and 5-year overall survival rates of 83%, 62%, and 50%, respectively, and disease-free survivals of 56%, 38% and 38% at 1, 3 and 5 years, respectively [94]. The time from diagnosis to LT appeared to have a high impact on survival rates, suggesting a natural selection of those tumors with more favorable characteristics. The authors suggested that a minimum of 12–24 months should be applied as a selection criterion during the evaluation for LT of these patients. In the recently published open label randomized controlled SECA-II trial, the application of more strict selection criteria led to a significant raise of overall survival after LT (1 and 5 years were 100% and 83%, respectively), with a median follow-up of 36 months (range 5–60 months) [100]. As a matter of fact, patients included in the SECA-II study showed better pre-LT prognostic factors and more favorable tumor biology (lower number of metastatic lesions, size of largest liver lesions, CEA levels, and recurrence risk scores) than SECA-I patients. Nevertheless, the burden of the disease was considerable at the time of LT in both cohorts.

Even though patients that were included had different tumoral characteristics and underwent different treatments before LT, it is becoming clear that it represents a valid option for curative intent in the context of colorectal liver metastases, offering the possibility of long-term overall survival to highly-selected patients with extensive disease. Data and experience are still limited, however several clinical trials are coming through (NCT 04161092, NCT 03494946, NCT 04616495, NCT 04874259). The major aim is to refine selection criteria in order to raise overall survival rates close to those of patients undergoing LT as a standard of care. Moreover, one multicentre Italian trial based in Milan (NCT 03803436) is aiming to assess the efficacy of LT compared with a matched cohort of patients included in another trial involving chemotherapy plus anti-EGFR. Among others, another element which needs to be evaluated is the possibility to add adjuvant chemotherapy after LT, considering possible graft toxicity. In this regard, the TRANSMET Trial from France (NCT 02597348) is currently comparing the survival rates between standard of care chemotherapy and LT plus adjuvant chemotherapy [101]. Another major challenge is related to the paucity of liver grafts, challenging the approval of new oncological indications. The recruitment of patients in LT trials is currently limited to a minimum percentage of the total amount of transplants per center, aiming to not impact the waiting time for other waitlisted patients. One of the suggested strategies is the use of marginal grafts, as patients with CRC rarely present at LT with either portal hypetension, end stage liver disease or deterioration of other organs functionality and thus could be more easily matched with these donors [100]. Another alternative to increase the donor pool is represented by the RAPID-protocol, which involves a two-stage hepatectomy followed by LT using a left-lateral split graft and delayed total hepatectomy [102]. Three trials, one in our center (NCT04865471), one in Oslo, Norway (NCT 02215889) and one in Jena, Germany (NCT 03488953) are currently evaluating this option. Additionally, a protocol started in Toronto (Canada), is also evaluating the option of living donor LT in patients with CRC (NCT 02864485). 

## 5. Cholangiocarcinoma and Liver Transplantation

Cholangiocarcinoma (CCA) is one of the most frequent primary liver cancers, second only to HCC. It can be classified in three subtypes: intrahepatic (iCCA), perihilar (pCCA) and distal extrahepatic (eCCA) cholangiocarcinoma. In all three subtypes the current gold standard for treatment is surgical resection [103]. Extensive surgery protocols have been established [104], however radical surgical resection can be achieved in <50% of patients due to insufficient remnant liver and difficulties in vascular reconstructions [104]. However, in many cases, complete surgical resection cannot be achieved [105]. Moreover, CCA frequently presents with local vascular infiltration, and primary sclerosing cholangitis (PSC) associated CCA is often considered as unresectable due to the underlying liver disease and predisposition to skip lesions [106]. Radiological treatments, such as intra-arterial therapies, ablation, radioembolization and brachytherapy (iodine-125 seed implantation) are used as locoregional oncological, palliative, and bridging to surgery in patients with unresectable or recurrent CCA after hepatectomy [107,108], however with no curative potential. Within this frame, [106]. LT has been suggested as a potential curative treatment for those patients presenting with non-resectable pCCA and more recently for patients with “very early” iCCA, since it ideally allows radical resection and eradication underlying PSC when associated. Patients included in clinical trials were free from extrahepatic metastases, vascular or lymphnodes invasion. Even though the initial experiences discouraged many centers to pursue this goal [109], a few groups kept on offering LT to these patients, improving selection criteria and treatment protocols. Eventually some impressive survival data were produced leading to internationally re-evaluate LT as a curative option [109] as a treatment for nonresectable CCA, particularly in those countries suffering from graft shortages. 

### 5.1. Intrahepatic Cholangiocarcinoma (iCCA)

ICCA is a subtype of CCA that arises from the intrahepatic biliary tract, which can be divided into mass-forming, periductal-infiltrating, intraductal, and undefined subtypes, depending on macroscopic growth patterns. The incidence of iCCA has been increasing in the last decades, particularly among cirrhotic patients [110]. Despite surgical advances, long-term outcomes of liver resections remain poor, with a 5-year overall survival of 40% and very high prevalence of postoperative morbidity [111,112,113]. Additionally, in >50% of patients the disease recurs, typically within 24 months after resection [114]. Even though iCCA is still widely considered a contraindication to LT, there is still a quite high percentage of grafts showing incidental tumors at explant pathology (1–3.3% of all LT) [115,116], as it still represents a diagnostical challenge. Retrospective data from these accidentally transplanted iCCA demonstrated an acceptable 5-year overall- and recurrence free-survivals in cirrhotic patients with “very early” iCCA (<2 cm), [117,118,119]. This led to reconsider LT as a potentially curative option in this context. Moreover, the 2 cm cut-off has been challenged by De Martin et al. who showed comparable survival rates after LT for iCCA of <2 cm and those of 2.1–3 cm [120]. As a matter of fact, in this study the only independent variable associated with tumoral recurrence was its differentiation, which, when available, reduces the impact of tumor size for prognosis. On the other hand, iCCA features are still often underestimated during pre-LT diagnostic evaluation, leading to higher recurrence rates and worse post-LT survival when compared to HCC [121]. Thus, careful consideration of potential higher aggressiveness of the tumor needs to be born in mind, especially in the context of PSC. Independent predictors of post-LT recurrence and survival include microvascular, perineural or lymphovascular invasion, multifocality, poor differentiation, infiltrative subtype, lack of neo- or adjuvant treatments [120,122]. In the noncirrhotic population, Lunsford et al. recently showed a 50% recurrence-free survival, and 83% 5-year overall survival following LT in six patients with locally advanced, unresectable iCCA [123]. Inclusion criteria were solitary tumor >2 cm or multifocal disease confined to the liver without evidence of macrovascular or lymph node involvement and sustained response to neoadjuvant gemcitabine-based chemotherapy. Neither tumor volume nor multifocality affected the incidence of disease recurrence after LT. This neoadjuvant protocol has also been proven successful in downstaging iCCA to surgical resections, which still remains the gold standard of treatment for iCCA. In this scenario, LT could be kept as an option to those remaining non-resectable despite the neoadjuvant treatment [124]. Validation of these findings in ongoing clinical trials may change the current exclusion of patients with iCCA from transplant programs, and the identification of the best selection criteria could further implement long term results (NCT 04195503, NCT 02878473, NCT04556214). For now, iCCA remains a contraindication for LT outside of clinical trials. Ongoing studies are evaluating the role of mutations in KRAS, fibroblast growth factor receptor and VEGF expression and dysregulated immune checkpoints [124]. Therefore it has been proposed to sequence the whole genome to facilitate the individuation of new therapeutic targets.

### 5.2. Perihilar Cholangiocarcinoma (pCCA)

PCCA is a subtype of CCA that arises anywhere from the second-order biliary ducts to above the site of cystic duct origin; it can have exophytic (mass-forming) and intraductal growth patterns. PCCA is one of the current challenges for hepatic surgery, which still represents the first-line treatment for this malignancy in case of localized disease [125]. Achievement of curative intent often requires implementation of surgery with neo- or adjuvant chemo and radiotherapies [126,127]. Still <20% of pCCA are amenable of surgery at diagnosis, due to the innate propensity of this tumor for the invasion of the adjacent vessels. For non-resectable patients, chemotherapy offers a minimal extension of survival which, in any case, remains <1 year [128,129], with a progression-free survival of 5 months [130,131]. Additionally, frequently pCCA arises from underlying PSC, which limits the possibility of resection [132]. LT has been considered as it can theoretically maximize resection margins and remove the underlying parenchymal liver disease when present. The first reports in the 1990s gave disappointing 28% 5-year survival and a 51% recurrence rate with LT alone, which withheld transplant centers from accepting this as [132]. LT can theoretically maximize resection margins and cure the underlying parenchymal liver disease. The early experience in the 1990s with LT alone gave disappointing 28% 5-year survival and a 51% recurrence rate for pCCA, which deterred transplant centers from accepting this oncological indication [122]. However, in these studies no selection criteria was imposed, treatment arms did not distinguish between pCCA and iCCA and neo- or adjuvant treatment where not included in the treatment protocols. Therefore, despite these dismal initial results, the University of Nebraska and the Mayo Clinic kept on working on this program until they established a successful multimodality protocol for unresectable pCCA preliminary to LT [133,134,135]. The so called “Mayo Clinic Protocol” includes external beam radiation, combined with intravenous 5-fluorouracil, followed by intraluminal brachytherapy and oral capecitabine. After this a routine exploratory laparoscopic staging is performed to confirm the absence of extrahepatic disease lymphonodal localizations prior to LT. By adopting this protocol, Heimbach et al. were able to obtain a 5-year survival exceeding 80% after LT in those patients with solitary tumors including nonresectable ones <3 cm in radial diameter not extending below the cystic duct, without evidence of lymph node metastases, and in those pCCA associated to PSC [135]. Similar results were confirmed in 12 large-volume transplant centers in the US, which reported a median 5-years disease-free survival rates of 65% [135]. Similar results were confirmed in 12 large-volume transplant centers in the US, which reported 5-years disease-free survival rates of 65% [136]. During the years it became evident that those patients with early disease had improved outcomes following LT [137,138,139], and that neoadjuvant chemoradiotherapy followed by LT offered the best outcomes for patients selected following the Mayo Clinic criteria [140,141,142]. Moreover, Mandel et al. demonstrated that patients selected for LT with these criteria reach a significant better survival compared to those not respecting them (59% versus 21% at 5-years) [143]. Thus, the “Mayo Clinic Protocol” has been gradually adopted, confirming 5-year survival rates of approximately 65–70% across different transplant centers [136,144,145,146]. These results, similar to those obtained in patients transplanted for HCC, led the United Network for Organ Sharing (UNOS) to allow the assignment of a Model for End-Stage Liver Disease (MELD) score exception to patients with unresectable pCCA or arising in the setting of PSC, for accessing LT [136,147]. However, concerns regarding organ allocation, waiting times, and the intensity of the neoadjuvant protocol have been limiting the spread of this indication in clinical practice. As for now, the guidelines of the European Association for the Study of the Liver recommend LT for pCCA to be limited to centers with clinical research protocols employing strict selection of patients and adjuvant or neoadjuvant therapy [10]. Still several patients drop out from the waiting list due to tumoral progression and its related complications, positive laparoscopy or inability to tolerate chemotherapy prior to LT. As demonstrated by a recent observational study, the estimated 82% 5-year survival rate precipitated to 58% on intention to treat analysis, since 46% of patients initially included did not access LT due to neoplastic progression [148]. The Mayo Clinic group reported that the risk factors for dropout of the LT waiting list due to disease progression were: CA 19.9 ≥500 U/mL, a mass ≥3 cm, malignant brushing or biopsy and a MELD score ≥20. Likewise, predictors of recurrence after LT were elevated CA 19.9, invasion of portal vein and evidence of residual tumor at explant [149]. Furthermore, the time interval between neoadjuvant therapy and LT was found to be inversely proportional to recurrence rates, which in turn correlates with tumor biology [150]. However, waiting time needs to be balanced with more pronounced fibrosis induced by prolonged radiotheraphy, hampering both staging laparoscopy and LT. Living donor LT could theoretically avoid timing issues. However ethical concerns toward living donation need to be considered as well at this stage of evidence. Ongoing and future studies will probably better address these issues and further refine treatment protocols [151]. A group at Washington University are currently recruiting for a prospective study which aims to assess whether highly selected patients still require neoadjuvant chemoradiation (NCT 00301379). Moreover, the use of sirolimus will be explored in a pilot trial (NCT 01888302). The rationale of giving sirolimus with gemcitabine and cisplatin is that it may be useful for patients with high risk of CCA recurrence after LT or either surgery. The results of these and other ongoing trials (NCT01549795, NCT02178280, NCT 04378023, NCT02232932) will be of great interest.

## 6. Conclusions

The LT scenario is undoubtedly evolving rapidly, with a plethora of new indications that could give hope for a better life for a large number of patients. However, these newer indications increase the pressure in an already difficult context of organ shortage. Strategies are therefore needed to increase the pool of transplantable organs that aim to ensure the balance between new indications and available resources. Moreover, it is mandatory to optimize the patients’ selection criteria to guarantee transplant advantages and achieve adequate patient and graft survival. Specialized surgeons, oncologists, hepatologists and radiologists should collaborate in a multi-disciplinary transplant team to ensure proper work-up and minimize the risks for these patients. Finally, the new scenario of transplants makes it essential to review and standardize the allocation systems and ethical considerations across countries to ensure the same treatment options for all patients.
